# Utility of imaging modalities for predicting carcinogenesis in lobular endocervical glandular hyperplasia

**DOI:** 10.1371/journal.pone.0221088

**Published:** 2019-08-15

**Authors:** Makiko Omori, Tetsuo Kondo, Hikaru Tagaya, Yumika Watanabe, Hiroko Fukasawa, Masataka Kawai, Kumiko Nakazawa, Akihiko Hashi, Shuji Hirata

**Affiliations:** 1 Department of Obstetrics and Gynecology, Faculty of Medicine, University of Yamanashi, Yamanashi, Japan; 2 Department of Pathology, Faculty of Medicine, University of Yamanashi, Yamanashi, Japan; University of Nebraska-Lincoln, UNITED STATES

## Abstract

**Objectives:**

To investigate the use of imaging methods for predicting carcinogenesis in lobular endocervical glandular hyperplasia (LEGH).

**Methods:**

We retrospectively analyzed preoperative images on transvaginal sonography and magnetic resonance imaging (MRI) in 23 cases with histologically diagnosed LEGH.

**Results:**

Shape of cervical multicystic lesions on MR images could be divided into two types the flower-type with many small cysts surrounded by larger cysts, and the raspberry-type with many tiny, closely aggregated cysts. Six (46%) of 13 cases had raspberry-type lesions that were not detected on transvaginal sonography but were seen on MRI. Adenocarcinoma in situ (AIS) was identified in 4 postmenopausal women with raspberry-type lesions during the follow-up periods. In these cases, cytologic examination by targeted endocervical sampling using sonography enabled early detection of AIS.

**Conclusions:**

MRI and cytologic examination by targeted endocervical sampling may be very useful for predicting carcinogenesis in LEGH.

## Introduction

Effective cervical cancer screening programs have led to reductions in the incidence of invasive squamous cell carcinoma of the uterine cervix. However, there has been no reduction in the incidence of invasive adenocarcinoma [1–3]. The detection of early cervical adenocarcinoma is an important task; however, its subtle cytologic features make early detection on Papanicolaou (Pap) smear tests challenging [4,5]. Furthermore, a subset of cervical adenocarcinoma is negative for high-risk human papillomavirus (HPV) DNA tests, and its precursor lesions have not been clearly defined [6,7].

A gastric type mucinous carcinoma, accounting for more than 20% of all cervical adenocarcinomas in Japan, carries a worse prognosis compared to the usual type endocervical adenocarcinoma and is reportedly negative for high-risk HPV [8–10]. Lobular endocervical glandular hyperplasia (LEGH) is a benign hyperplastic lesion of endocervical glands that exhibits gastric differentiation [11–14]. Case reports of adenocarcinoma in situ (AIS)/adenocarcinoma coexisting with LEGH and several other genetic studies suggest that a subset of LEGH may be a precursor to some cervical adenocarcinomas including gastric type mucinous carcinoma and minimal deviation adenocarcinoma [14–21]. LEGH is also negative for high-risk HPV, as we have previously reported [13,14].

Patients with LEGH often have watery to mucoid vaginal discharge and multicystic lesions found characteristically in the upper part of the cervix on transvaginal sonography and magnetic resonance imaging (MRI) [14,22,23]. The histologic appearance of LEGH is lobular proliferations of benign-looking endocervical glands surrounding a larger gland. These glands are lined by columnar cells possessing pyloric gland-type mucin that is immunopositive for HIK1083 and MUC6 [11,12,14,24]. Pyloric gland-type mucin is yellowish on a conventional Papanicolaou (Pap) smear and can be confirmed by the HIK1083-labeled latex agglutination test (HIK1083 latex test) performed on the cervical discharge [21,22,24–26].

Few studies have investigated how the appearance of LEGH lesions may change in different imaging modalities during carcinogenesis [27]. In the present study, we retrospectively investigated the utility of transvaginal sonography and MRI for predicting carcinogenesis in LEGH.

## Materials and methods

### Case selection

The present study was performed in accordance with the Declaration of Helsinki and the Ethical Guidelines for Medical and Health Research Involving Human Subjects (Ministry of Health, Labour and Welfare, Japan. https://www.mhlw.go.jp/file/06-Seisakujouhou-10600000-Daijinkanboukouseikagakuka/0000080278.pdf), and was approved by the Human Ethics Review Committee of Yamanashi University Hospital (No.1925). The requirement for written informed consent was waived by “the University of Yamanashi Personal Information Protection Personal Information Regulations”. We extracted 34 consecutive cases of LEGH with/without malignancy that were histologically confirmed by hysterectomies at the Department of Obstetrics and Gynecology, Yamanashi University Hospital, Yamanashi, Japan between January 2001 and March 2018. We excluded 5 cases that showed malignancy on Pap smear tests and/or colposcopically directed biopsies at the initial visits, and excluded 6 cases that did not receive MRI examination. Finally, we applied 23 cases into this study, and retrospectively analyzed clinical findings, cytologic findings, and images.

All patients received Pap smear tests and transvaginal sonography at the initial visit.

When LEGH was considered clinically, the patients received the HIK1083 latex tests and MRI examination within 1 month of the initial visit. Thirteen patients underwent hysterectomies within 3 months of the initial visit because of the LEGH diagnosis. Whereas 10 patients were followed by transvaginal sonography and cytologic examination every 3 to 6 months because they wished for monitoring rather than immediate surgery. After continued monitoring and follow-up, 4 women received hysterectomies due to AIS arising in LEGH, 4 women due to LEGH, 1 woman due to uterine leiomyoma, and 1 woman due to ovarian cystadenoma. The 10 patients underwent 3 or more transvaginal sonography, 6 patients of which underwent 2 or more MRI examinations during follow-up periods.

The patients’ median age at the initial visit was 55 years (range, 37–79 years). The cases consisted of 3 nulliparous women and 20 parous women (para 1, 6 patients; para 2, 12 patients; and para > 3, 2 patients). Furthermore, 11 women were premenopausal and 12 were postmenopausal (Table 1).

**Table 1:** Relevant clinical and imaging data in the case series.

Case	Age	G	P	Menopause	Watery D	AEC-GAM	HIK test	Cervical multicystic lesion			Malignancy
	(years)							Sonography	MRI		
									Type	Size (mm)^b^	
1	37	3	2	Pre	+	+	+	+	Flower	35×35×32	−
2	38	3	1	Pre	−	+	+	−	Raspberry	21×4×16	−
3	39	1	1	Pre	+	+	+	+	Flower	23×22×26	−
4	39	1	1	Pre	+	+	+	+	Flower	35×22×27	−
5	45	1	0	Pre	+	+	+	+	Flower	28×28×39	−
6	46	2	2	Pre	+	+	+	+	Flower	31×37×41	−
7	47	2	2	Pre	−	+	+	+	Flower	33×36×37	−
8	51	2	2	Pre	+	+	+	+	Flower	23×22×21	−
9	51	5	1	Pre	+	+	+	−	Flower	30×24×26	−
10	51	2	2	Pre	+	+	+	+	Flower	39×44×44	−
11	52	2	2	Pre	+	+	+	+	Flower	22×16×15	−
12	55	2	2	Post	−	+	+	+	Raspberry	19×9×15	AIS
13	57	3	2	Post	+^a^	+	+	+	Raspberry	18×10×12	−
14	58	3	3	Post	+^a^	+	+	−	Raspberry	7×2×8	−
15	59	2	2	Post	+	+	+	−	Raspberry	5×8×6	−
16	60	2	0	Post	−	+	+	+	Raspberry	23×14×12	−
17	62	4	2	Post	−	+	+	−	Raspberry	10×5×6	−
18	64	7	4	Post	−	+	+	+	Raspberry	12×7×14	AIS
19	65	2	1	Post	+^a^	−	+	−	Raspberry	20×12×13	−
20	70	1	1	Post	+	+	+	+	Raspberry	30×17×22	AIS
21	71	3	2	Post	−	+	+	−	Raspberry	16×15×16	−
22	73	2	2	Post	+^a^	+	+	+	Raspberry	18×16×17	−
23	79	1	0	Post	+	+	+	+	Raspberry	16×10×10	AIS

^a^This case had hydrometra in addition to watery to mucoid vaginal discharge at the initial visit.

^b^Size is measured as height×anteroposterior diameter ×transverse diameter on MRI.

AEC-GAM, atypical endocervical cells with gastric-type mucin on conventional Papanicolaou smear; Age, age at the initial visit; AIS, postoperative histologically diagnosed adenocarcinoma in situ; Duration, duration to hysterectomy; Flower, flower-type appearance; G, gravida; HIK test, HIK1083-labeled latex agglutination test; MRI, magnetic resonance imaging; P, para; Post, postmenopausal state; Pre, premenopausal state; Raspberry, raspberry-type appearance; Size, size on MR images; Sonography, on transvaginal sonography; and Watery D, watery to mucoid vaginal discharge.

The HIK1083 Latex Kit (Kanto Chemical, Tokyo, Japan) was used to detect the expression of pyloric gland-type mucin in cervical mucus obtained by cotton swab, according to the instructions provided by the manufacturer.

### Imaging tests

We performed MRI examinations using the 1.5 Tesla SIGNA Excite (GE Healthcare, Milwaukee, WI, US) and analyzed axial and sagittal T2-weighted images (T2WI) and axial and sagittal contrast-enhanced fat-suppressed T1-weighted images (T1WI). Two diagnostic radiologists in our hospital made the diagnoses. Two diagnostic radiologists or one gynecologist measured the cervical multicystic lesions as height × anteroposterior diameter × transverse diameter on sagittal and axial images of the T2WI. In the present study, a second gynecologist, blinded to the previous results, reexamined all lesions. If the two results were discordant, a third gynecologist was consulted.

The multicystic lesions were measured as height × anteroposterior diameter on the sagittal sonographic image. After making several measurements during a single examination, we extracted the most accurately-measured data.

### Cytologic examinations

**Fig 1:**
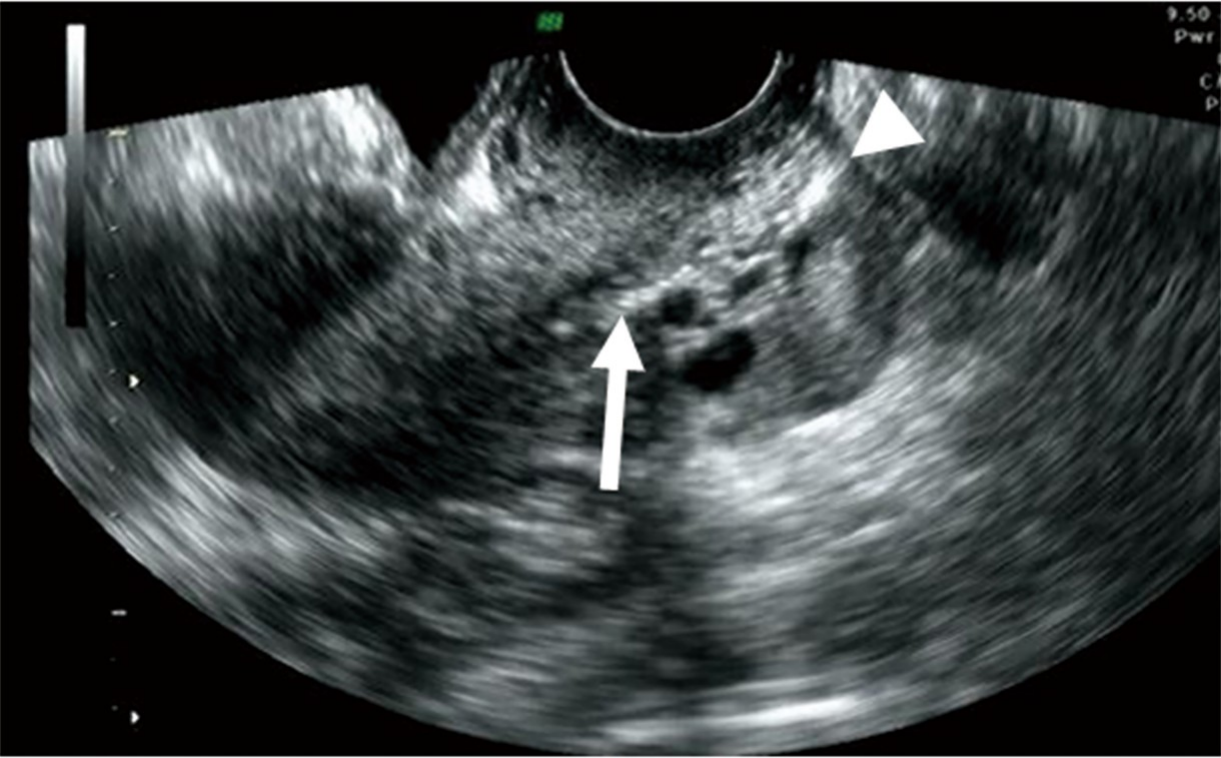
Targeted endocervical sampling. The hyperechoic line indicates the site of cytologic sampling in the cervical canal (arrow head, external cervical os; arrow, upper end of the sampling site) (transvaginal sonography).

When LEGH was suspected, we obtained cytological specimens by “targeted endocervical sampling” because adenocarcinoma associated with LEGH typically occurs in the upper part of the endocervical canal and may be difficult to reach by routine cervical sampling. At first, we measured the length between the internal cervical os or the top of the multicystic lesion and the external cervical os on transvaginal sonography. We then inserted a sampling device into the cervical canal based on the measured length. We often used an endometrial sampler marked with measurement tick marks that was easy to insert into the cervical canal. After sampling, we confirmed success by looking for a hyperechoic line that reflected the sampling sites in the cervical canal (Fig 1). We used the Cytobrush Plus (CooperSurgical, Trumbull, CT, US), the Endocyte (Laboratoire, Paris, France), the Screebrush (Soft Medical, Tokyo, Japan), or the Softcyte (Soft Medical, Tokyo, Japan) endometrial samplers. The last three are devices for making endometrial smears. All cytologic smears were prepared by conventional methods.

When endocervical cells with yellowish mucin were observed on a Pap smear, we described it as atypical endocervical cells with gastric-type mucin (AEC-GAM) in addition to the original cytological diagnosis [21]. Furthermore, we diagnosed it as high-grade when the AEC-GAM had irregular three-dimensional arrangements of the nuclei and altered mucin that was faintly yellow, or orange mucin localized on the apical side, or varicolored mucin of pink, orange, and yellow.

### Statistics

For statistical analyses, we used the Fisher's exact test and the Mann-Whitney U test with *p* values of less than 0.05 considered statistically significant.

## Results

Sixteen (70%) of 23 women presented with watery to mucoid vaginal discharge. The premenopausal women presented with a larger amount of watery to mucoid discharge than the postmenopausal women. Four postmenopausal women (17%) additionally presented with hydrometra on sonography. Twenty-two cases (96%) showed AEC-GAM on cytological examination. All cases (100%) were positive on the HIK1083 latex tests (Table 1).

Transvaginal sonography revealed cervical multicystic lesions in 16 cases (70%), while the lesions were found in all cases on MR images. MRI revealed multiple cysts located predominately in the upper part of the cervix. They were hyperintense on T2WI and hypointense on contrast-enhanced fat-suppressed T1WI. The median maximum diameter of the multicystic lesions on MRI was 22 mm (range 8–44 mm). The maximum diameter and the volume (height × anteroposterior diameter × transverse diameter) were larger in premenopausal cases than in postmenopausal cases (the maximum diameter: premenopausal cases, median 35 mm, interquartile range (IQR) 25–38 mm vs. postmenopausal cases, median 17 mm, IQR 13–19 mm, *p* = 0.0003 and the volume: premenopausal cases, median 21 cm^3^, IQR 12–42 cm^3^ vs. postmenopausal cases, median 2.4 cm^3^, IQR 1.0–3.9 cm^3^, *p* = 0.0006, respectively) (Table 1). We detected no obvious solid components in any cases. Retained secretion showing hyperintensity on T2WI was occasionally found in the cervical canal and the vaginal fornix.

**Fig 2:**
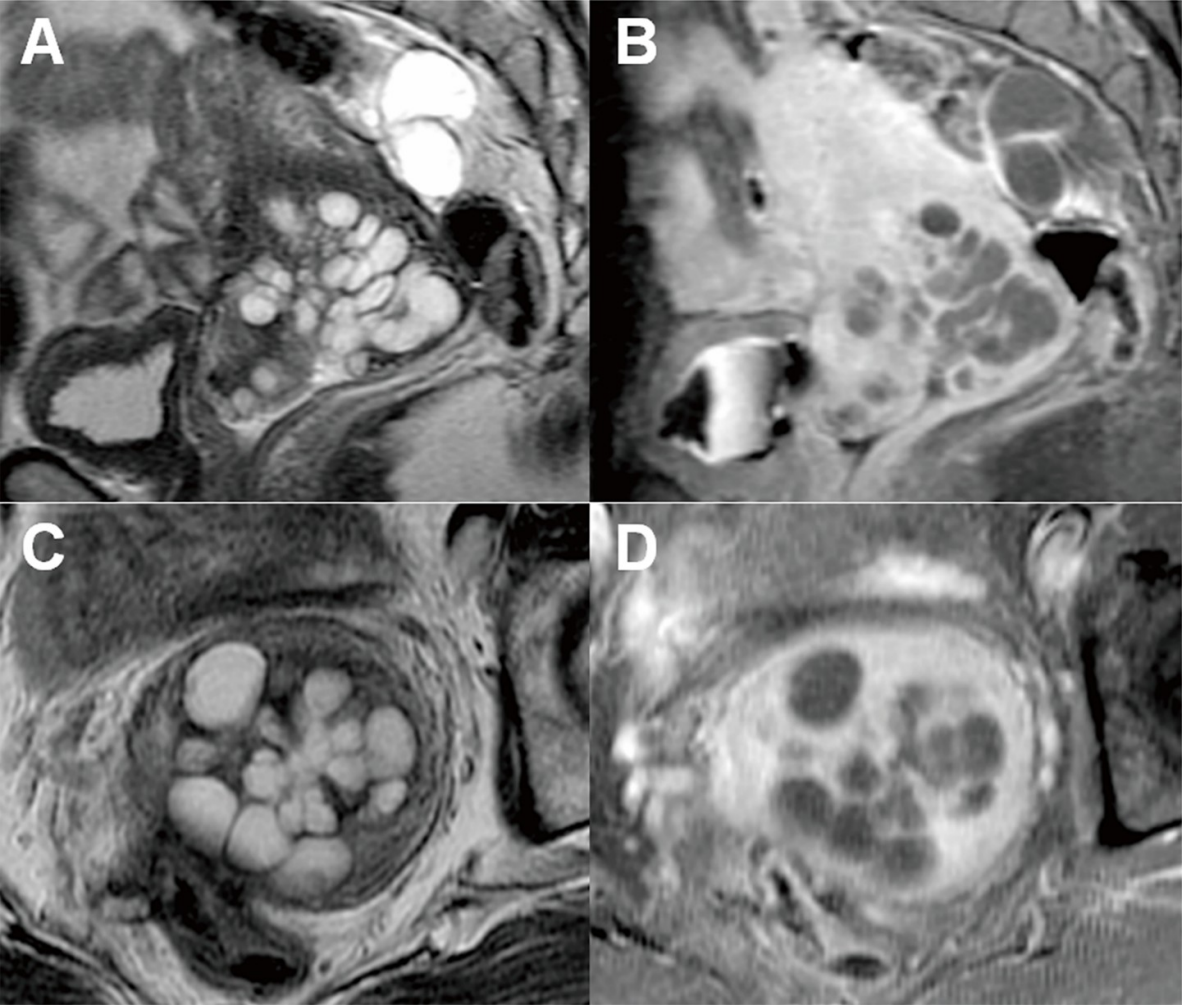
Flower-type multicystic lesion of the cervix. (Case 10) Magnetic resonance images show a lesion of many small cysts surrounded by many larger cysts with hyperintensity on T2-weighted images and hypointensity on contrast-enhanced fat-suppressed T1-weighted images (A and B, sagittal images; C and D, axial images; A and C, T2-weighted imaging; and B and D, contrast-enhanced fat-suppressed T1-weighted imaging).

**Fig 3:**
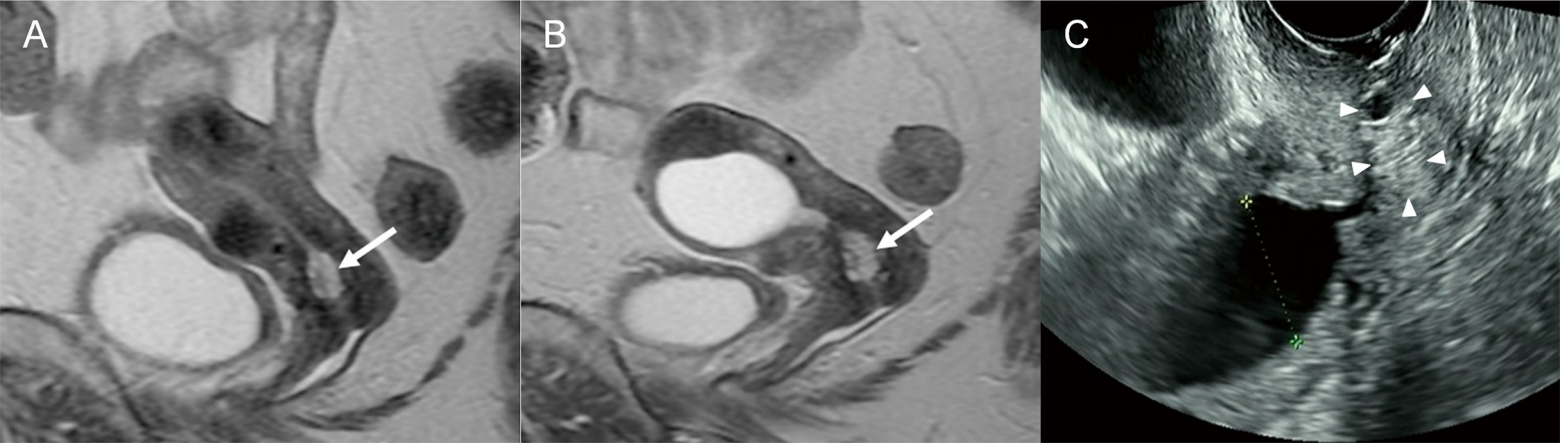
Raspberry-type multicystic lesion of the cervix. (Case 18) **A.** Magnetic resonance (MR) image at the initial visit shows a lesion of numerous, closely aggregated tiny cysts (arrow) with hyperintensity on T2-weighted images. **B.** MR image before hysterectomy shows hydrometra in addition to a raspberry-type multicysytic lesion (arrow) with little change in size and shape. **C.** Transvaginal sonography shows hydrometra and a hyperechoic lesion that includes a small cyst (arrow heads) in the endocervix.

The shape of the multicystic lesions on MR images could be divided into two types. The “flower-type” lesion consisted of a large number of small cysts surrounded by many larger cysts. Takatsu, et al. referred to this type of lesion as a “cosmos sign” [23] (Fig 2). The “raspberry-type” lesion was a close aggregation of numerous tiny cysts with occasional small cysts (Fig 3). The patients’ age was significantly younger in the flower-type group than in the raspberry-type group (flower-type group, median 47 years, IQR 41–51 years, vs. the raspberry-type group, median 62 years, IQR 58–70 years, *p* = 0.0006). We observed the flower-type cystic lesion in 10 (91%) and the raspberry-type cystic lesion in 1 (9%) of the premenopausal cases. However, all 12 (100%) of the postmenopausal cases had the raspberry-type lesion, a statistically significant difference (*p*<0.0001). The raspberry-type lesion was smaller than the flower-type lesion (the maximum diameter: the raspberry-type lesion, median 18 mm, IQR 14–20 mm vs. the flower-type lesion, median 35 mm, IQR 27–39 mm, *p* = 0.0002, and the volume: the raspberry-type lesion, median 2.2 cm^3^, IQR 1.2–3.8 cm^3^ vs. the flower-type lesion, median 26 cm^3^, IQR 15–43 cm^3^, *p* = 0.0001, respectively).

We could not detect 1 (10%) of the 10 flower-type lesions and 6 (46%) of the 13 raspberry-type lesions during transvaginal sonography at the initial visit because of their small size (Table 1). However, re-evaluation by sonography revealed only a few small cysts or a small hyperechoic lesion containing a few small cysts in these cases.

### Follow-up study

**Fig 4:**
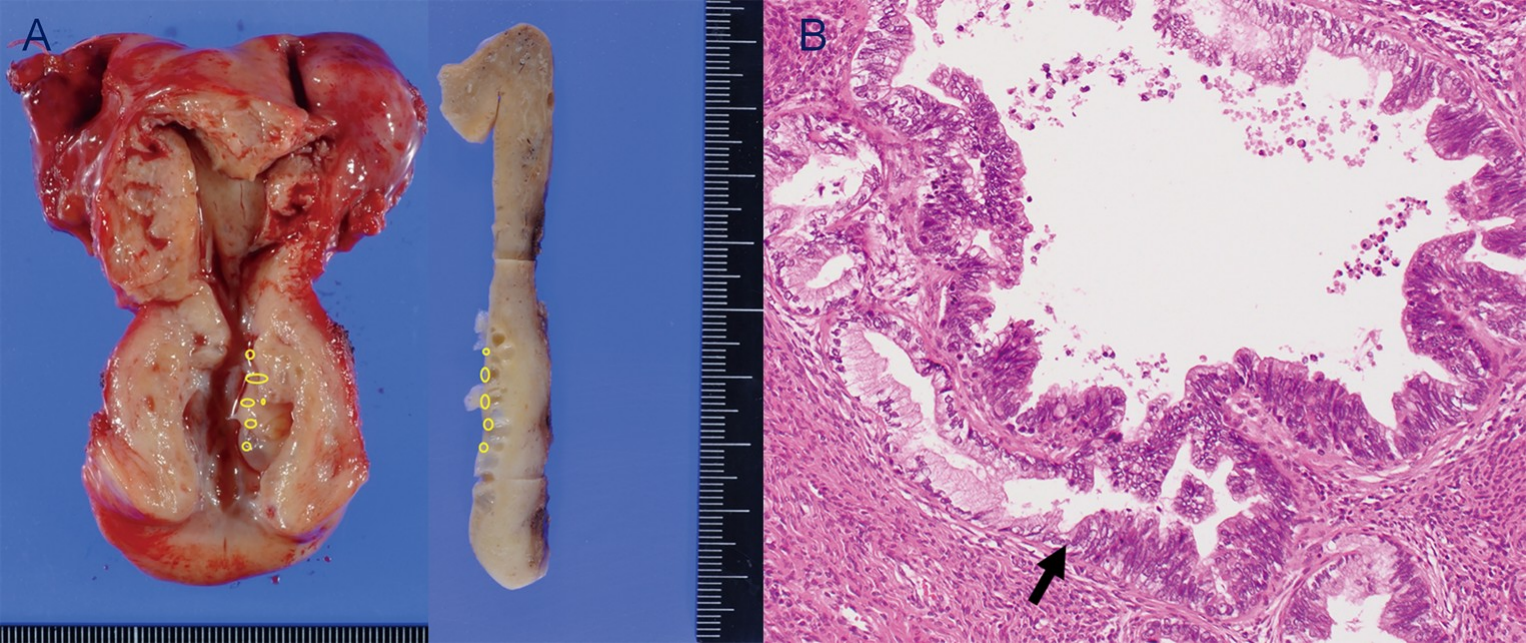
Macroscopic findings and histologic findings. (Case 18) **A.** The surgically resected uterus shows watery to mucoid secretion within the cervical canal, a lesion that includes multiple small cysts in the endocervix. The cut section shows many small cysts lining the inner side of the endocervix. AIS was identified as a skip lesion in the superficial area of the LEGH lesion (open circle). **B.** Histologic findings show adenocarcinoma in situ coexisting with lobular endocervical glandular hyperplasia. Front formation (arrow) between obvious atypical cells and non-atypical columnar cells is observed in the gland with lobular endocervical glandular hyperplasia without stromal invasion (H&E, ×20).

We evaluated changes in multicystic lesions in patients that underwent 3 or more transvaginal sonography and/or 2 or more MRI examinations (Table 2). This corresponded to 10 cases that underwent hysterectomy after a median follow-up period of 17 months (range 7–57 months). Postoperative histologic examination revealed AIS associated with LEGH in 4 cases (the AIS-LEGH group) and LEGH without malignancy in 6 cases (the LEGH group). The AIS lesions were sporadically detected in the superficial small glands of LEGH in the cervical canal near the isthmus in all 4 cases (Fig 4).

**Table 2:** Follow-up cases.

Case	Age	Multicystic lesion						
No.	(years)	Sonography		MRI			Duration	Malignancy
		Initial (mm)^a^	Before Hys (mm)^a^	Type	Initial (mm)^b^	Before Hys (mm)^b^	(months)	
3	39	22×18	28×18	Flower	23×22×26	n.p.	19	−
4	39	28×22	not available	Flower	35×22×27	n.p.	27	−
6	46	37×26	36×26	Flower	31×37×41	n.p.	8	−
10	51	40×27	45×34	Flower	39×44×44	48×53×53	15	−
14	58	not detected	not detected	Raspberry	7×2×8	n.p.	7	−
22	73	18×14	18×17	Raspberry	18×16×17	12×16×16	57	−
12	55	14×14	18×10	Raspberry	19×9×15	12×8×15	14	AIS
18	64	14×9	12×10	Raspberry	12×7×14	12×7×14	7	AIS
20	70	18×22	21×22	Raspberry	30×17×22	31×16×21	41	AIS
23	79	12×8	10×9	Raspberry	16×10×10	12×5×10	51	AIS

^a^Size is measured as height×anteroposterior diameter on sagittal image of transvaginal sonography.

^b^Size is measured as height×anteroposterior diameter×transverse diameter on MRI.

Age, age at the initial visit; AIS, postoperative histologically diagnosed adenocarcinoma in situ; Duration, duration to hysterectomy; Before Hys, size of multicysytic lesion before hysterectomy; Flower, flower-type appearance; Initial, size of multicystic lesion at the initial visit; MRI, magnetic resonance imaging; n.p., not performed; Post, postmenopausal state; Raspberry, raspberry-type appearance; and Sonography, transvaginal sonography.

The clinical courses of representative cases are described below. Case 4, a 39-year-old woman who wished to preserve her fertility, presented with profuse watery to mucoid vaginal discharge and a large flower-type cystic lesion. The patient underwent conization with a histologic diagnosis of LEGH. However the lesion was too large for complete resection by conization. Nineteen months after the initial visit, she delivered with a caesarean section due to cephalopelvic disproportion. At 27 months after the initial visit, she experienced profuse vaginal discharge and underwent simple hysterectomy. Her final histologic diagnosis was LEGH with no malignancy. In Case 10, a 51-year-old premenopausal woman, both sonography and MRI showed that her flower-type cystic lesion had increased in size after 15 months of monitoring, but the postoperative histological diagnosis revealed no atypia.

Cases 12, 18, 20, and 23 had raspberry-type lesions and developed AIS during the follow-up period. All 4 cases showed high-grade AEC-GAM. We identified these cells on targeted endocervical cytology before clusters of AIS appeared. Cases 12 and 18 had no symptoms at the initial visits. However, Case 12 had watery to mucoid vaginal discharge and a high-grade AEC-GAM 9 months later. Case 18 had high-grade AEC-GAM 3 months after presentation and hydrometra 5 months later (Images 3B, 3C). At 14 months and 7 months after their initial visits, Case 12 and Case 18, respectively, were cytologically diagnosed with AIS and received hysterectomies. Case 20 and Case 23 had watery to mucoid discharge and were diagnosed with high-grade AEC-GAM at 27 months and 32 months after the initial visit, respectively. At 41 months and 51 months after the initial visit, respectively, they were cytologically diagnosed with AIS and received hysterectomies. During the follow-up period of Case 20, the size of each small cyst decreased and the density of the cystic lesion increased, but the size of the entire lesion did not change. The other 3 cases of AIS showed no significant changes of the multicystic lesions. Cases 12, 18 and 23 were negative for high-risk HPV testing prior to hysterectomy, and Case 20 did not take HPV testing.

There was no difference between the AIS-LEGH group and the LEGH group in regards to change in total lesion size seen on sonography (the maximum diameter: AIS-LEGH group, median 93%, IQR 85–107% vs. LEGH group, median 106%, IQR 99–116%, *p* = 0.57 and the volume: AIS-LEGH group, median 94%, IQR 93–101% vs. LEGH group, median 124%, IQR 115–131%, *p* = 0.057, respectively). There was also no difference in shape change on MR images between the 2 groups from initial visit to hysterectomy.

## Discussion

A subset of LEGH have been shown to develop adenocarcinoma including gastric-type mucinous carcinoma [14–21]. However, its risk factor has not been clearly elucidated. LEGH and adenocarcinoma associated with LEGH are high-risk HPV-negative endocervical lesions [9,13,14]. The vaccine for HPV does not interfere the genesis of these adenocarcinoma, and their early detection is still a challenge. It would be beneficial for decision management if the clinical signs of LEGH carcinogenesis could be predicted.

A multicystic lesion located in the upper part of the cervix is the most characteristic finding of LEGH [14,23]. Detecting a cervical cystic lesion on sonography often leads to diagnosis of LEGH. The MR images of the multicystic lesion typically show a large number of small cysts surrounded by many larger cysts, which is called a “cosmos sign” [23,28,29]. The present study revealed that shape and size of the lesion depended on menopausal status and age. We determined there were two types of multicystic LEGH lesions that could be seen on MR images. One was a flower-shaped lesion as described above that was commonly seen in premenopausal women. The other was a smaller lesion displaying a close aggregation of numerous tiny cysts resembling a raspberry that was commonly seen in postmenopausal women.

There are few studies investigating how the sonographic and MR images of LEGH may change as they develop adenocarcinoma. Ando, et al. reported an increase in tumor size of LEGH with atypia [27]. In our cases of AIS arising in LEGH, the cystic lesions seen on sonography and MRI did not change significantly in size or shape, although in one case, the cysts’ density increased in a raspberry-type lesion. Adenocarcinoma arising in LEGH usually presents with solid components in multicystic lesions on MR images [23,28–30]. However, we did not see solid components in our cases of AIS arising in LEGH.

All of our patients that developed AIS associated with LEGH were postmenopausal and presented with a raspberry-type cystic lesion on MR images. Detecting a raspberry type cystic lesion in a postmenopausal woman can be considered a significant finding, and an appropriate management plan should be initiated.

However, it should be noted that the raspberry-type cystic lesions were not be recognized as multicystic lesions on sonography in 6 (46%) of 13 cases. In these cases, re-evaluation by sonography showed only a few small cysts or a hyperechoic lesion containing a few small cysts located in the upper part of the cervix. Whereas MRI revealed even small raspberry-type cystic lesions. MRI appears to be an important diagnostic modality for LEGH when there are raspberry-type cystic lesions that may be related to carcinogenesis.

We may see hydrometra in postmenopausal cases of LEGH when there is a large amount of secretion from a LEGH lesion that is located at the characteristic site near the isthmus and there is age-related stenosis of the cervical canal. If hydrometra is observed on sonography, it is important to look for a cystic lesion in the upper portion of the cervix. The HIK1083 latex test is helpful for confirming that the lesion exhibits gastric phenotype, as we have previously reported [22]. Although watery to mucoid vaginal discharge and hydrometra are not related to carcinogenesis of LEGH, a sudden increase of watery to mucoid vaginal discharge and sudden occurrence of hydrometra may provide helpful information as to possible changes in biologic behavior of the LEGH lesion.

It is extremely important to identify atypical cells on cytologic smears for the management of LEGH. LEGH and AIS/adenocarcinoma associated with LEGH typically occur in the upper part of the cervix [14,23]. Unfortunately, it is difficult to obtain the endocervical specimen in that portion of the cervical canal with only a commonly used cervical sampler. “Targeted endocervical sampling,” as described above, allowed us to obtain appropriate cytologic specimens from the entire cervical canal including near the isthmus. In our cases, we identified AIS as a skip lesion in the superficial area of the LEGH lesion, but not in larger glands of the deep part. These clusters of AIS could be reliably captured by targeted endocervical cytology.

We did not determine the time span LEGH requires to develop cancer. In the present study, the longest period between initial visit and developing AIS was 51 months. We also found a significant difference in age of approximately 13 years between women with AIS/adenocarcinoma associated with LEGH (median age 67 years) and women with LEGH without malignancy (median age 54 years) [21]. Some cases of LEGH will develop cancer relatively slowly. Thus, once LEGH is detected, continuous follow-up is needed to detect early adenocarcinoma.

The present study has several limitations. First, the number of subjects was small, and the follow-up period was short for some cases of LEGH without AIS. Second, the reference bias may have occurred because all patients made the gold standard for the final diagnosis. Third, investigation of asymptomatic group with cervical multiple cysts will be needed to avoid bias in data interpretation. Fourth, gynecologists will need to learn the procedure for targeted endocervical sampling in order to use this method of diagnosis and to follow LEGH progression.

A raspberry-type multicystic lesion on MR images in postmenopausal women may be associated with carcinogenesis in LEGH, but these small lesions may not be visible on sonography. Seeing several small cysts or hyperechoic lesions, including a few small cysts located near the isthmus on sonography, will indicate a multicystic lesion of LEGH. In conclusion, MRI examination will be useful for risk stratification of LEGH. When a raspberry-type multicystic lesion is detected, detailed cytological examination is recommended.
